# Effects of Input Parameter Range on the Accuracy of Artificial Neural Network Prediction for the Injection Molding Process

**DOI:** 10.3390/polym14091724

**Published:** 2022-04-23

**Authors:** Junhan Lee, Dongcheol Yang, Kyunghwan Yoon, Jongsun Kim

**Affiliations:** 1 Department of Mechanical Engineering, Dankook University, Yongin 16890, Korea; junhan0526@dankook.ac.kr (J.L.); didehdclf2@naver.com (D.Y.); 2 Molding & Metal Forming R&D Department, Korea Institute of Industrial Technology, Bucheon 14442, Korea; 3 SimTech, Bucheon 14442, Korea

**Keywords:** injection molding, artificial neural network (ANN), quality prediction, linear regression, polynomial regression, nonlinearity

## Abstract

Artificial neural network (ANN) is a representative technique for identifying relationships that contain complex nonlinearities. However, few studies have analyzed the ANN’s ability to represent nonlinear or linear relationships between input and output parameters in injection molding. The melt temperature, mold temperature, injection speed, packing pressure, packing time, and cooling time were chosen as input parameters, and the mass, diameter, and height of the injection molded product as output parameters to construct an ANN model and its prediction performance was compared with those of linear regression and second-order polynomial regression. Following the preliminary experiment results, the learning data sets were divided into two groups, i.e., one showed linear relation between the mass of the final product and the range of packing time (linear relation group), and the other showed clear nonlinear relation (nonlinear relation group). The predicted results of ANN were relatively better than those of linear regression and second-order polynomial for both linear and nonlinear relation groups in our specific data sets of the present study.

## 1. Introduction

Injection molding is a representative plastic molding technique that can rapidly produce products with complex shapes, which require precise dimensions, in large batches. This technique is widely used in various fields, ranging from household goods to the automotive industry or electronic and electrical industries [1,2]. It is the process of molding a product by injecting a plastic resin melted at a high temperature into a space with a specific shape within a mold at high speed and pressure. Thus, the rheological behavior and state of the material during molding are affected by input process conditions, i.e., the melt and mold temperatures, injection speed, packing pressure, and packing time. Those five variables ultimately affect the final product quality, i.e., mass or dimensions of the final product. The fabrication of a product with the desired quality requires specific combinations of input process conditions. If the input process conditions are unsuitable, defects such as short shots or flash may occur. However, because the plastics used in the injection molding process have highly complex thermo-viscoelastic behavior, it is difficult to realize and maintain the desired quality [3]. In production sites where the injection molding process is applied, a trial-and-error approach has been frequently used to explore process conditions through references or guidebooks. It requires significant time and cost, as well as involving high uncertainty because it depends heavily on the experience of molding workers. To resolve these issues, the computer aided engineering (CAE) technique has been applied as a very useful pretest tool that can model the relationship between injection molding parameters and final quality and optimize the process [4,5,6]. However, the injection molding simulation analysis utilizing the CAE consumes significant time when estimating the final dimensions, in addition to the many assumptions concerning the material properties [3]. Furthermore, despite the increased accuracy of recent CAE analysis, it is well known that there have been differences between the predicted and actual values of output quality due to the inherent nonlinear and viscoelastic characteristics of plastic resins.

Thus, there has been a consistent need for a new and improved method for optimally manufacturing injection molded products with a targeted output, such as mass or specific lengths. In response to this demand, there has recently been an increasing amount of research applying artificial neural network (ANN) technology to model and optimize the relationship between input variables, such as melt and mold temperatures, and output variables, such as mass or specific length in the injection molding process [7,8,9,10,11,12,13,14,15,16]. The ANN, currently the most promising language in the artificial intelligence (AI) field, is a well-known and representative technique exhibiting powerful and practical performance in identifying relationships that contain complex nonlinearities [3]. Ozcelik et al. [7] constructed an ANN with a multi-input single-output (MISO) structure, in which five multi-input parameters, i.e., melt temperature, mold temperature, packing pressure and time, and cooling time, were set to perform an injection molding experiment. The warpage of a molded product was measured at a specific location, which was chosen as the targeted output (single output). They conducted and demonstrated the usefulness of the MISO structure to predict the molding conditions for minimizing the amount of warpage at specific locations. Yin et al. [8] set the same five input parameters and obtained the warpage information of the automobile glove compartment cap through CAE results data rather than actual experiments. Similar to Ozcelik et al. [7], they constructed an ANN by applying the MISO structure and verified whether the amount of warpage was the minimum through actual experimentation by predicting the process conditions that minimize warpage. Yang et al. [9] set 10 process conditions as input parameters and built an ANN structure that predicts the mass of the injection molded product as an output parameter with MISO. They also conducted a study to determine the optimal set of process conditions for molding a product with a targeted mass. Their prediction showed good results while the relationships between input and output parameters were almost linear. Lee et al. [10] applied shape information such as volume and area for multiple molds in addition to the usual six process conditions as input parameters to predict the mass of a product for an arbitrary mold. The ANN was built by using experimental data and CAE analysis data. Based on the ANN model, they obtained good results by building a system for deriving the combination of input parameters that can be applied to molds of arbitrary shapes. Gim et al. [11] measured the cavity pressure and time using sensors. Then, five specific points, i.e., start point of filling stage, switchover point, maximum point of cavity pressure, packing endpoint, and cooling endpoint, were selected to extract pressure and time values and used as input parameters. ANN structure that predicts the mass of the injection molded product (spiral) as an output parameter with MISO. In addition, they conducted research on optimizing the molding window through sensitive analysis and obtained good results. Recently, as in the studies of Abdull et al. [12] and Heinisch et al. [13], research on multi-input multi-output (MIMO) structured ANNs is being actively performed to predict multiple target qualities from multiple process conditions. Table 1 lists studies in which ANNs are applied to the injection molding process.

In Table 1, previous studies used various shapes ranging from simple to complex, such as automobile glove components or IC trays. It can be seen that the injection molding process shows good results by using a simple ANN model, even with a complex shape or data structure. However, the linear range was applied in the relationship between parameters on a case-by-case basis, and this may suggest the possibility that limited results were obtained. In addition, it can be confirmed that most of the previous injection molding studies applied by ANNs are relatively simple structures, including one or two hidden layers or a small number of neurons. Concerning the nonlinearities, the performance of the ANN model is closely related to the complexity of the model [18]. In general, as the complexity increases, the number of hidden layers and neurons increases. The more nonlinear and complex the relationship between input and output parameters expressed in the ANN is, the greater the complexity required for the ANN model, and the problem may not be solved with a small number of hidden layers and neurons. Thus, when the number of hidden layers or neurons of the constructed ANN model is small, it can be considered a simple physical system with relatively strong linearity in the relationship between input and output parameters. Gim et al. [11], Abdul et al. [12], Heinisch et al. [13], Ke et al. [14], and Yang et al. [17] used an ANN model with one hidden layer for analyzing their specific data, while Ozcelik et al. [7], Yin et al. [8], Yang et al. [9], Lee et al. [10] and Huang et al. [15] used a model with two hidden layers. This means that the relationship between input and output parameters in the injection molding process can exhibit strong linearity. It can be possible to derive better results through other regression methods such as linear or polynomial. Therefore, to apply artificial neural networks to the injection molding process, it is necessary to check and exclude these possibilities. However, in previous studies on injection molding, confirmation and understanding of these problems were insufficient. In this respect, there have been studies comparing the performance of ANN with other regression analyses in the injection molding process. Heinisch et al. [13] set different methods for generating injection molding data groups and built ANN and Polynomial models to compare performance. After comparing the ANN and polynomial models, Heinisch et al. [13] concluded that they could not generally provide a guide regarding which method is better. These results are judged as the result of failing to represent the characteristics of each model by comparing the ANN and regression models only in the range where the relationship between parameters is linear.

Thus, preliminary experiments were performed to distinguish the data sets into linear and nonlinear groups. The range of packing time was chosen as an input parameter to determine the nonlinearity with the mass of the final product, a representative output parameter in preliminary experiments. In the data set of linear relation groups, the range of packing time was selected as 3.0~18.0 s, and the range of packing time was chosen as 3.0~30.0 s, including the packing time over 18.0 s for the data set of nonlinear relation group in the present study. Finally, in ANN modeling, linear and polynomial regressions were used for both linear and nonlinear groups to evaluate their respective accuracies and describe the strengths and weaknesses of each model.

## 2. Experiment

### 2.1. Material and Molding Equipment

**Figure 1:**
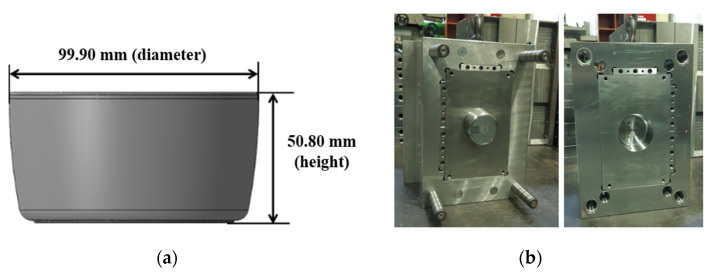
Images of (**a**) the rice bowl and (**b**) the mold.

In the present study, children’s tableware was selected as a target product, and a series of experiments were performed to obtain injection molding data. The target product was a bowl shape with a nominal diameter of 99.90 mm and a height of 50.80 mm, as shown in Figure 1a, and a two-stage mold with one cavity was utilized, as shown in Figure 1b. The hot runner system was applied to the mold, which is a direct system allowing the hot runner nozzle to be in contact with the center of the product. Polypropylene (PP) of LUPOL GP1007F (LG chemical) was used as the resin for product molding. The physical properties of LUPOL GP1007F described by the manufacturer are shown in Table 2. The 150-ton injection molding machine (LGEII-150, LSMtron) was used for the injection molding experiment. Table 3 shows the specifications of this injection molding machine.

### 2.2. Experimental Conditions

**Figure 2:**
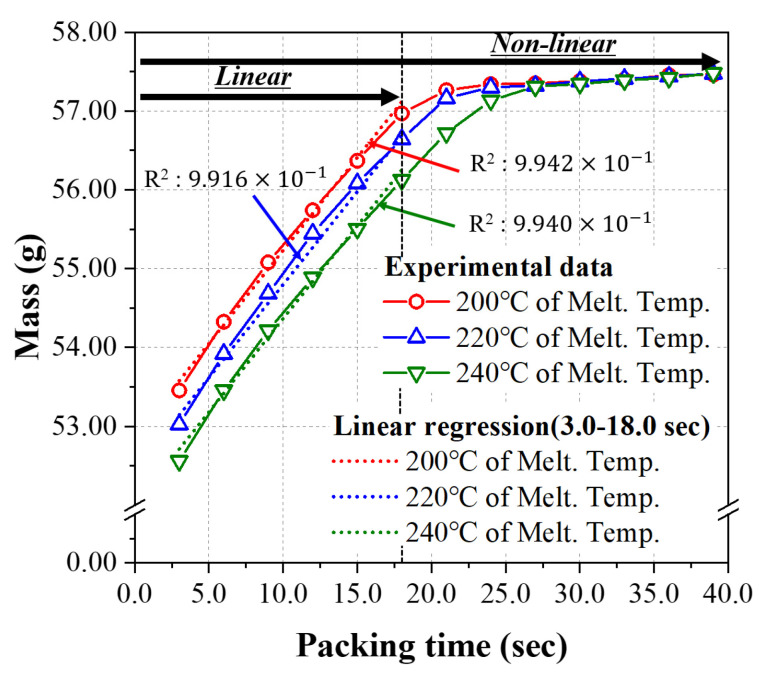
Results of the preliminary experiment to show linear and nonlinear relation between packing time and mass.

Based on the recommended conditions provided by the resin manufacturer and the database of Moldflow Insight 2021 (Autodesk), the melt temperature and mold temperature ranges were set in three levels for the injection molding experiment, as shown in Table 4. Furthermore, a series of preliminary experiments were performed to determine the process window of packing pressure and the range of packing time through which a normal product can be molded for the mold and product used in this study, and these were also applied in three levels. From the results of preliminary experiments, the performance of the ANN based on two groups of data sets was evaluated in the present study. The first group of data sets showed a strong linear relationship with an R^2^ score higher than 0.99 between the range of packing time (input parameter) and the mass of the molded product (output parameter). The second group of data sets showed a representative nonlinear relationship between them, as shown in Figure 2. The packing time of 6.0 s~18.0 s was applied to the data set of the linear relation group, as shown in Table 3, while the packing time of 3.0 s~39.0 s, including over 18.0 s, was applied to the data set of nonlinear relation group which will be shown in Table 5 later. The injection speed and cooling time were derived through CAE analysis using Moldflow Insight 2021, and the ranges of those process conditions were set at three levels, as are other conditions.

Table 5 lists 50 process conditions of above mentioned “linear relation group”. Based on the levels presented in Table 3, 27 combinations (experiment #1~#27) were created by the orthogonal array of L27, and 23 combinations (experiment #28~#50) were randomly generated within the corresponding range.

Table 6 shows the process conditions; the packing time of 3.0 s ~ 39.0 s was taken as the preliminary experiments to find the nonlinear characteristics between input and output parameters. For the conditions in Table 6, three different melt temperatures were applied for each data set. The melt temperature of 200 °C was applied to the experiments of #51~#63, where only the packing time varied from 3.0 s to 39.0 s with an interval of 3.0 s, while the other process conditions were kept constant. For the other two sets of experiments, the melt temperature of 220 °C and 240 °C were applied to experiments #64~#76 and #77~#89, respectively. The clear nonlinear relationship shown in Figure 2 is the results of these 3 sets of experiments shown in Table 6. The mass and the other two output parameters, i.e., the diameter and height of the final product, were measured and tested for nonlinear analysis of ANN in the present study.

### 2.3. Measurement of Product Qualities

**Figure 3:**
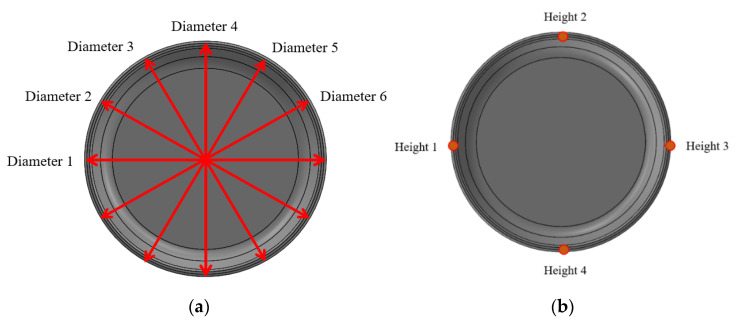
Measurement points for (**a**) diameter and (**b**) height.

To build an ANN prediction model, the mass, nominal diameter, and height of the injection molded product shown in Figure 1 were measured and considered as output parameters for each injection molding condition shown in Table 4 and Table 5. The mass of the injection molded product was measured by a CUX420H (CAS), a digital weighing scale, and the diameter of the product was determined by the average value of measurements at a total of six points shown in Figure 3a using the Datastar200 (RAM OPTICAL INSTRUMENT), a non-contact optical measuring instrument. The height of the product was determined by the average value of measurements at four points using the Mitutoyo Digimatic Height Gage, as shown in Figure 3b.

## 3. Building the Model to Predict the Product Qualities

### 3.1. Artificial Neural Network

**Figure 4:**
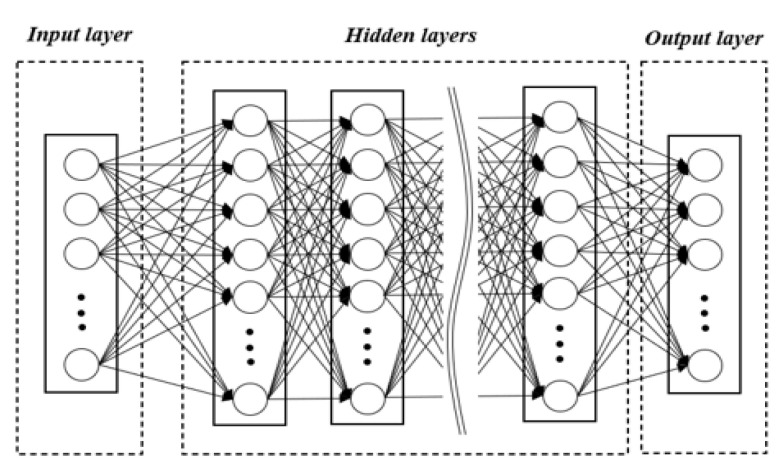
Schematic of the structure of the artificial neural network (ANN).

The ANN model mimics the process of the human brain recognizing and solving problems. As in the neural network constituting the human brain, this model has a computational processing structure in which neurons are arranged in each computation layer of the ANN. Figure 4 shows how this ANN structure is connected between input and output parameters. The ANN is an algorithm in which the structure is largely divided into input, hidden, and output layers, and the corresponding neurons are placed on each layer. In addition, a different number of neurons arranged on each layer can be set for each layer [19,20].

The back propagation algorithm is the most common learning method for training ANNs because the calculation and construction of this model are simple. The term “back propagation” refers to errors propagating in the opposite direction of the ANN’s progression. Errors are defined as the difference between output values of ANN and the actual values in the data set. The errors are used to calculate the changes in previous neurons in a backward direction. Thus, the back propagation algorithm requires input and output values of the training data, a method called supervised learning.

**Figure 5:**
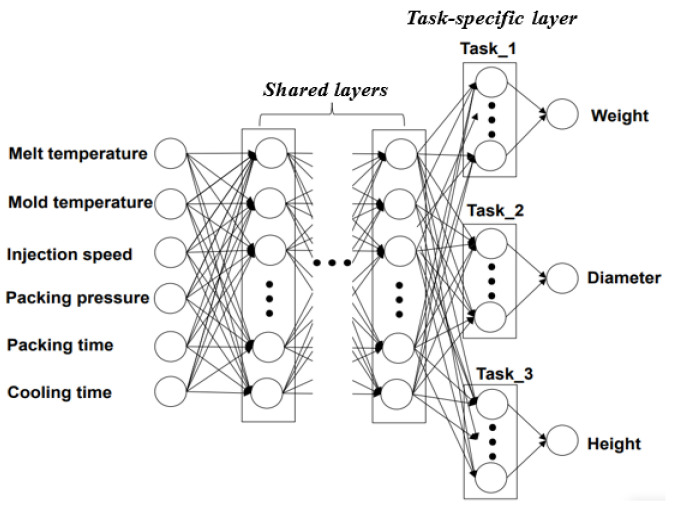
The multi-input multi-output (MIMO) structure using multi-task learning in the present study (hard parameter sharing) [21,22].

In the present study, an ANN with a MIMO structure was utilized to establish the relationship between multiple input parameters and multiple output parameters, as shown in Figure 5. Furthermore, the multi-task learning technique was applied to the typical MIMO structure shown in Figure 4 by assigning the task-specific layer for each output parameter [21,22]. Moreover, by placing the task-specific layer, which consists of one or more layers, for each output parameter, the root mean square error (RMSE) for each output parameter was individually calculated to minimize the summation of the RMSEs of all output parameters. The conventional MIMO method is known to be difficult to reflect the characteristics of each parameter accurately because the output parameters are related to each other, and all characteristics are learned dependently [22], so this study strived to resolve this issue by using the multi-task learning technique, as described well in other studies [23,24].

### 3.2. The Search for Optimal Hyper-Parameters

In training a machine learning model using an ANN, parameters that the user must set are called hyper-parameters. Because the initial setting of these parameters determines the efficiency and performance of the ANN, it is important to set the appropriate hyper-parameters according to the purpose of the ANN. Thus, the hyper-band technique [25] was used to determine the range of hyper-parameters, as shown in Table 7. This method is widely used because it requires significantly less time for optimization than conventional techniques, such as the grid search method, random search method, and Bayesian search method. It further showcases the excellent performance of the derived results.

## 4. Results

### 4.1. Injection Molding Experiment

**Figure 6:**
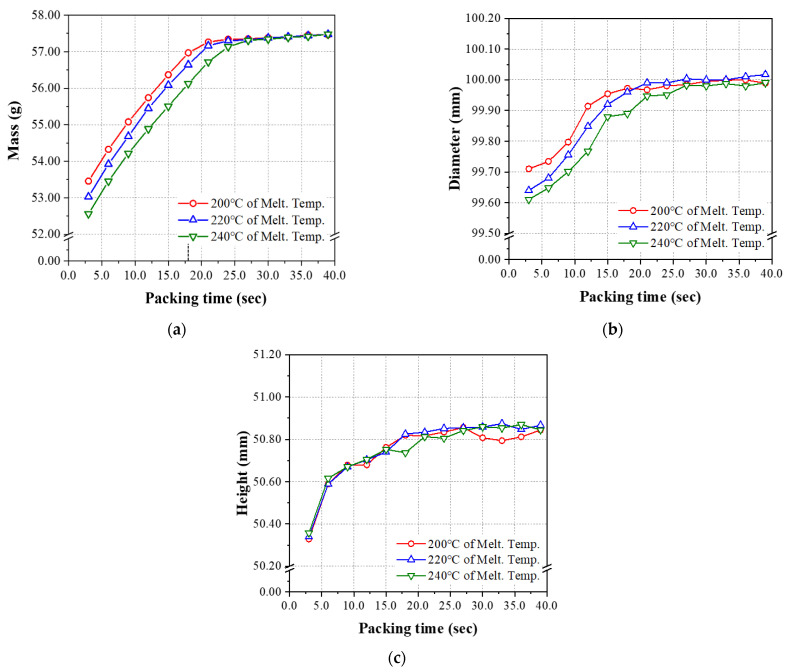
Experimental measurement results for the process conditions of Table 5 showing nonlinearity (packing time: 3.0~39.0 s): (**a**) mass, (**b**) diameter, and (**c**) height.

All the measurement results concerning the mass, diameter, and height of the final injection molded product are presented in Table A1 (Appendix A) for the case of the linear relationship between parameters with a packing time ranging from 6.0 s to 18.0 s. Table A2 (Appendix A) shows the injection molding experiments, in which the maximum value of the packing time was extended to 39.0 s among the injection molding conditions. According to the experimental data shown in Figure 6, when applying the packing time of 3.0~39.0 s, clear nonlinearity can be found in all the results of three output parameters, i.e., mass, diameter, and height. In particular, Figure 6a shows clear nonlinearity between the mass of the final injection molded product and packing time, as shown in the preliminary experiment in Figure 2. When the melt temperature was 200 °C, the linear and nonlinear sections were divided by the boundary with a packing time of 18.0 s.

Even though the linear relation was well suited to only one of the output parameters, i.e., the mass of the final product as shown in Figure 2, the packing time of 18.0 s was taken as a useful criterion. For convenience, the ANN model was constructed, and its performance was evaluated by dividing the experiments (or data sets) into two groups depending on the above-mentioned criterion, i.e., the packing time of 18.0 s in the present study. As shown in Table A1 and Table A2, the data groups were divided into a group with a packing time in the range of 3.0~18.0 s (linear relation group) and a group with an extended packing time ranging from 3.0 to 39.0 s (nonlinear relation group).

### 4.2. The Prediction Models Learned by the Linear Relationship Group (Packing Time ≤ 18.0 s)

In Table A1 and Table A2, a dataset with a packing time between 3.0 and 18.0 s was selected to form a linear relation group as mentioned above, and based on this, an ANN model was constructed. Fifty combinations from Table A1 and 18 combinations from Table A2 were selected to create a “linear relation group” with 68 combinations. Among the selected combination data, 54 combination datasets were used as training data for the ANN model. Seven of the remaining combination data (# 28, 33, 38, 43, 48, 55, 77) were used as validation data sets for the ANN model during training. The other seven combination data were used as test data to evaluate the prediction performance of the final ANN model. When the values of hyper-parameters in Table 8 searched by hyper-band technique were selected, the minimum RMSE value of output parameters could be obtained for the final ANN structure.

The performance of this ANN model was compared to those of a linear regression and a second-order polynomial regression model that used the same training data set calculated by the library (scikit-learn) in the Python package. As a final step, the test data set consisted of experiments #29, 34, 39, 44, 49, 56, and 78 that were applied to the constructed prediction models, and the experimental and prediction results are compared and summarized in Table 9. As seen in Table 9, the RMSE values for mass, diameter, and height of the ANN model were generally lower than those of the linear regression and second-order polynomial regression. We can conclude that the prediction performance of the final ANN model obtained for the linear relation group was relatively better than that of the linear regression and second-order polynomial regression models.

**Figure 7:**
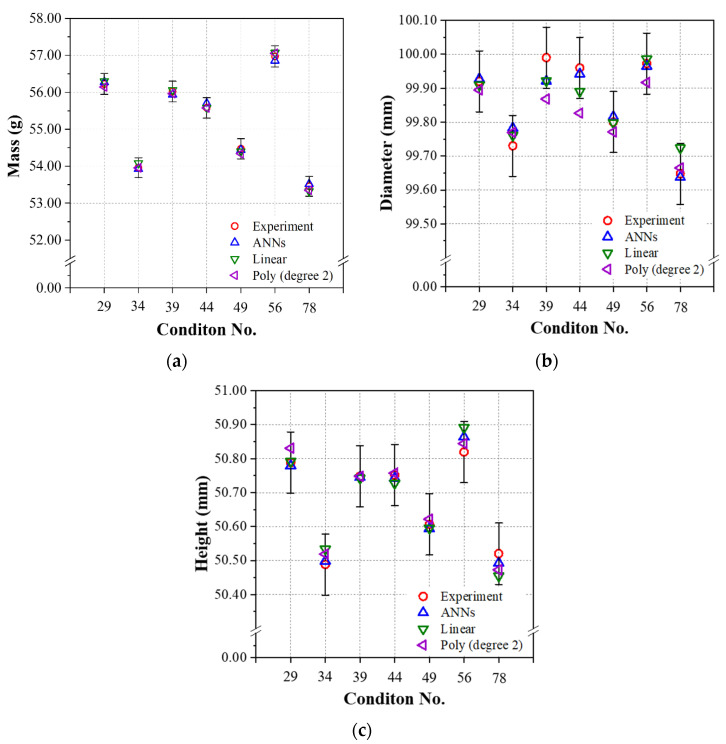
Performances of the prediction models using test data learned by the linear relation group (packing time was 3.0~18.0 s): (**a**) mass, (**b**) diameter, and (**c**) height.

Figure 7 shows the predicted results of three models obtained from the test data and experimental results with error bars calculated by applying ISO20457:2018 (Plastics molded parts—Tolerances and acceptance conditions); the dimensional quality standard for injection-molded products, as well as the mass quality standard for general PP. The calculated standard error equivalent to ISO20457:2018 of the injection molded product used in this study was ±0.009 mm [27] for both diameter and height, and ±0.5% [28] was applied as the standard error of the mass for PP molded product. According to Figure 7, both ANN and linear regression satisfied all the quality standards for the mass, diameter, and height of the present injection molded product. In contrast, in the case of the second-order polynomial regression, several combinations of experimental cases (#39, 44) failed to meet the quality standard for diameter, as shown in Figure 7b.

**Figure 8:**
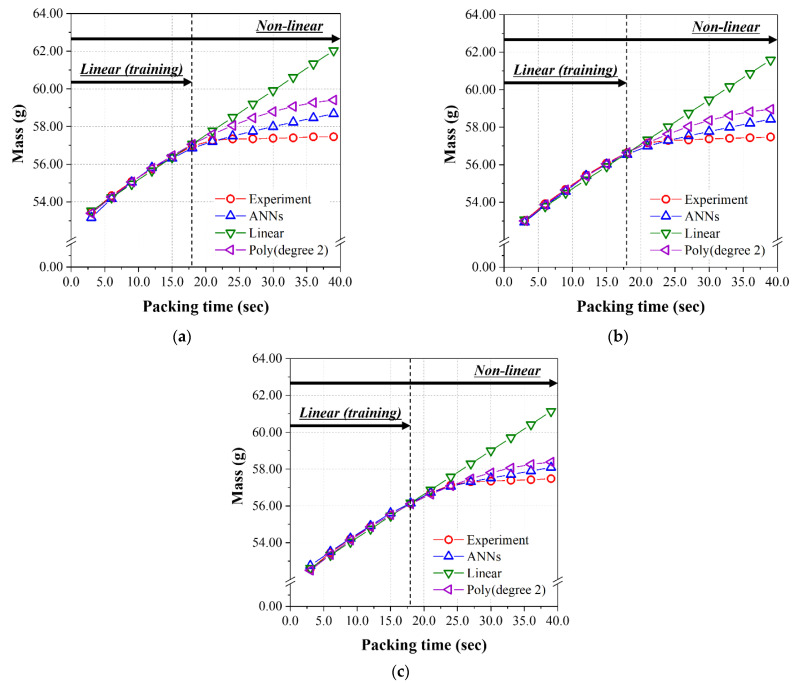
Predicted mass of nonlinear relation group from Table A2 using the models learned by the linear relation group (packing time was 3.0–18.0 s). Melt temperatures; (**a**) 200 °C, (**b**) 220 °C, and (**c**) 240 °C.

To find the relationship between the input and output parameters in a different way, the predicted results of three models were graphically shown for the data sets in Table A2 as a function of the packing time. Here, the previous test data set (#29, 34, 39, 44, 49, 56, and 78) was substituted for the data set in the linear range for packing time (#51~56, 64~69, 77~82). Figure 8a–c, which were performed at 200 °C, 220 °C, and 240 °C, respectively, show the prediction and experimental results of the final mass as a function of the packing time for the linear range of 3.0~18.0 s and extra range over 18.0 s.

**Figure 9:**
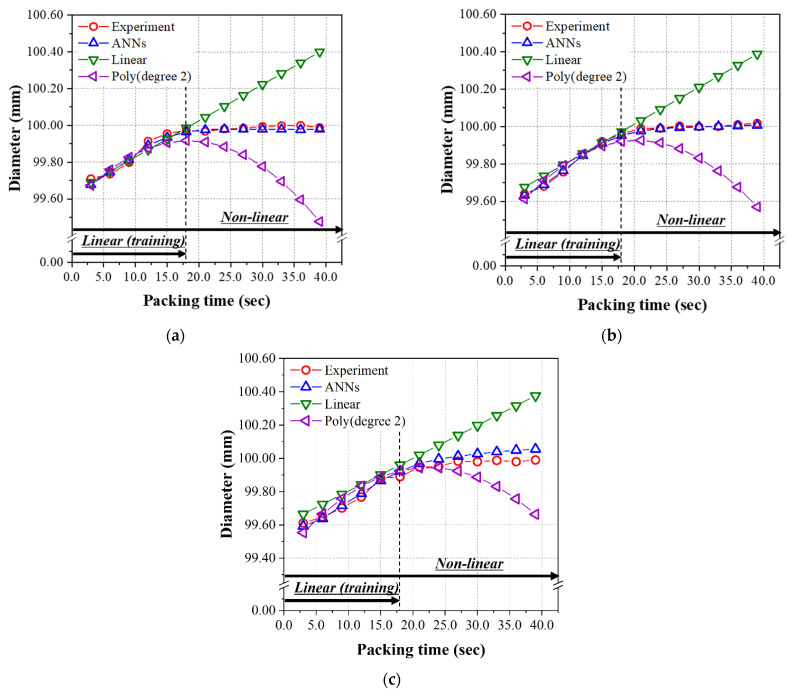
Predicted diameters of the nonlinear relation group from Table A2 using the models learned by the linear relation group (packing time was 3.0~18.0 s). Melt temperatures; (**a**) 200 °C, (**b**) 220 °C, and (**c**) 240 °C.

**Figure 10:**
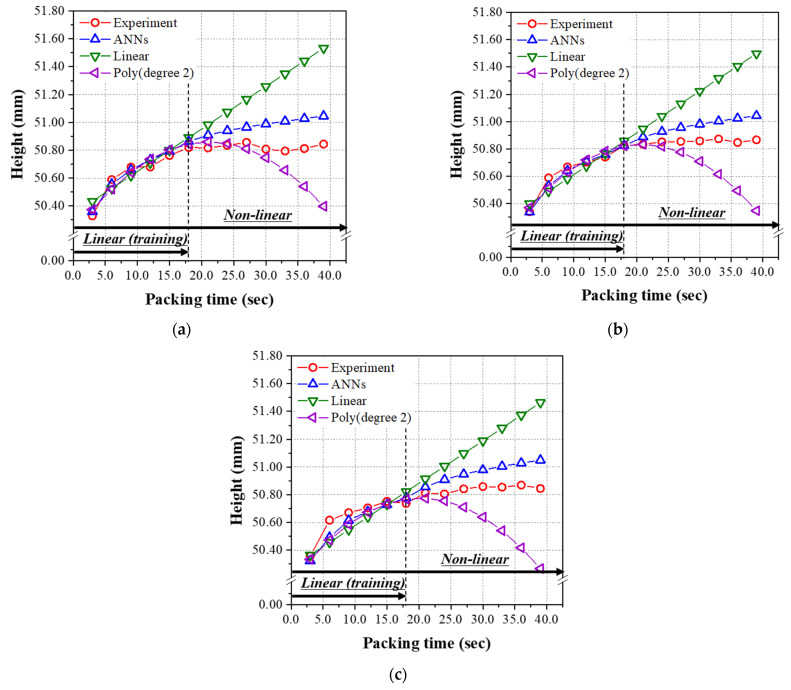
Predicted heights of the nonlinear relation group from Table A2 using the models learned by the linear relation group (packing time was 3.0~18.0 s). Melt temperatures; (**a**) 200 °C, (**b**) 220 °C, and (**c**) 240 °C.

As seen in Table 10, the RMSE values for the mass of all the three models, i.e., the ANN model, the linear regression, and second-order polynomial regression, are very low. Interestingly, the lowest RMSE was found for the case of second-order polynomial regression. The predicted and experimental data for the mass over 18.0 s are illustrated for reference. Similar to the results from the test data set given in Table 9, the ANN model has the minimum and lowest RMSE among those three models for the diameter and height shown in Table 10. Figure 9 and Figure 10 show the prediction and experimental results for diameter and height as a function of the packing time. From the results of the data set in the linear range for packing time (#51~56, 64~69, 77~82), the prediction performance of the final ANN model was excellent by comparing with that of the linear regression and second-order polynomial regression models.

### 4.3. The Prediction Model Learned by the Non-Linear Relationship Group

A total of 89 combination datasets shown in Table A1 and Table A2 were selected, and 71 combinations were used as training data for the ANN model. 9 of the remaining combinations data (# 28, 33, 38, 43, 48, 58, 70, 73 and 88) were used as validation data for the ANN model during training. The other 9 combination datasets (#29, 34, 39, 44, 49, 59, 71,74, and 89) were used as test data to evaluate the performance of the ANN model. When the hyper-parameter values in Table 11 searched by the hyper-band technique were selected, the minimum RMSE value of output parameters could be obtained for the final ANN structure in the same way as in the linear relation group.

Table 12 compares the RMSE values for the prediction results by applying the test data that were not used to construct the model. The RMSE values of the ANN model for all three output parameters were lower than those of the other models. From the results obtained for this nonlinear relation group, the prediction performance of the final ANN model was much better than that of the linear regression and second-order polynomial regression models.

**Figure 11:**
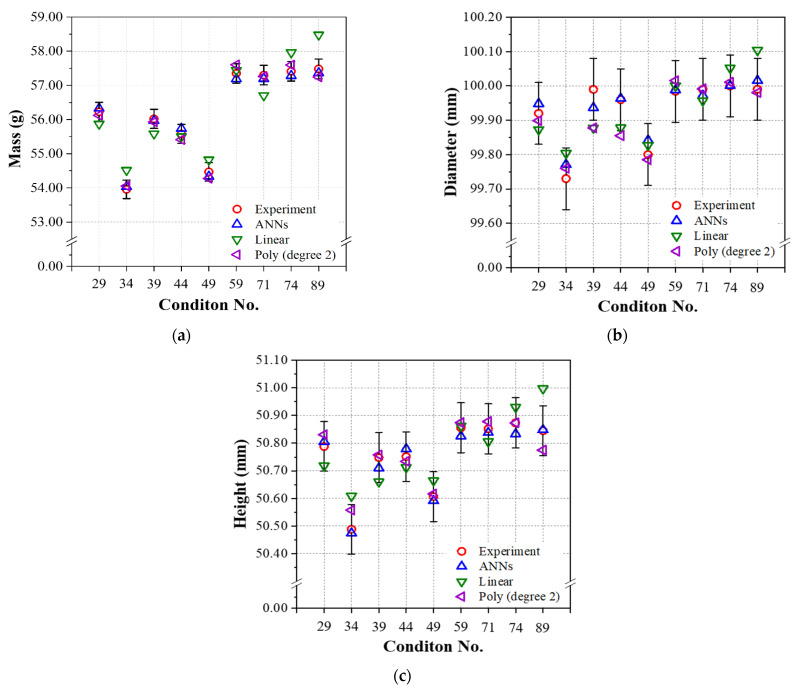
Performances of the prediction models using test data learned by the nonlinear relation group (packing time was 3.0–39.0 s): (**a**) mass, (**b**) diameter, and (**c**) height.

Figure 11 shows the predicted results of three models obtained from the test data and experimental results with error bars, as shown in Figure 7 for the linear relation group. All the predicted values of the ANN satisfied the standard specifications. In contrast, the predicted values of the linear regression tended to deviate from the quality standard, and there was a significant deviation or error from the experimental value. The predicted values of the second-order polynomial regarding the mass, diameter, and height of the injection molded product tended to be located within the quality standard. In contrast, only two combination datasets (#39, 44 in Figure 11b) deviated from the quality standard.

**Figure 12:**
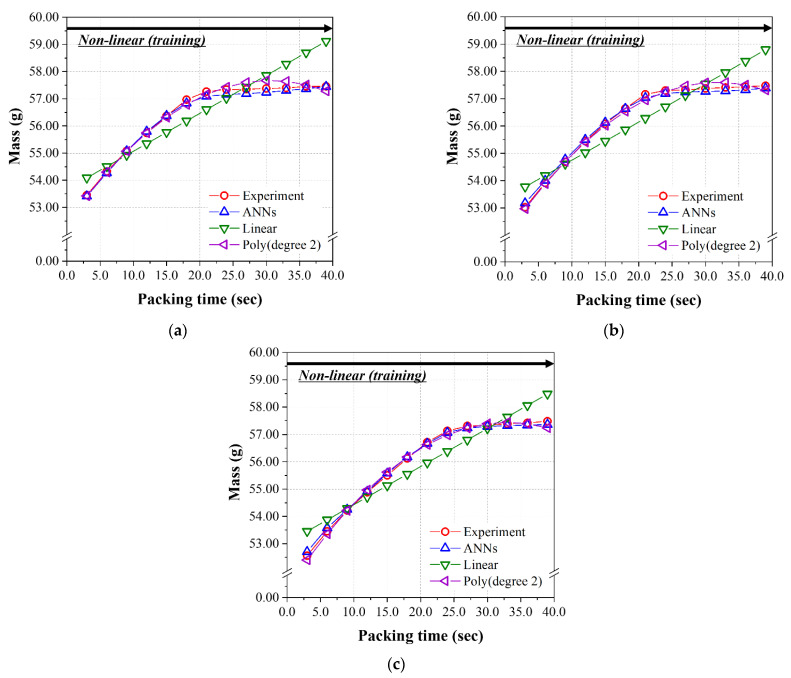
Predicted mass of the nonlinear group from Table A2 using the models learned by the nonlinear group (packing time was 3.0–39.0 s). Melt temperatures were (**a**) 200 °C, (**b**) 220 °C, and (**c**) 240 °C.

**Figure 13:**
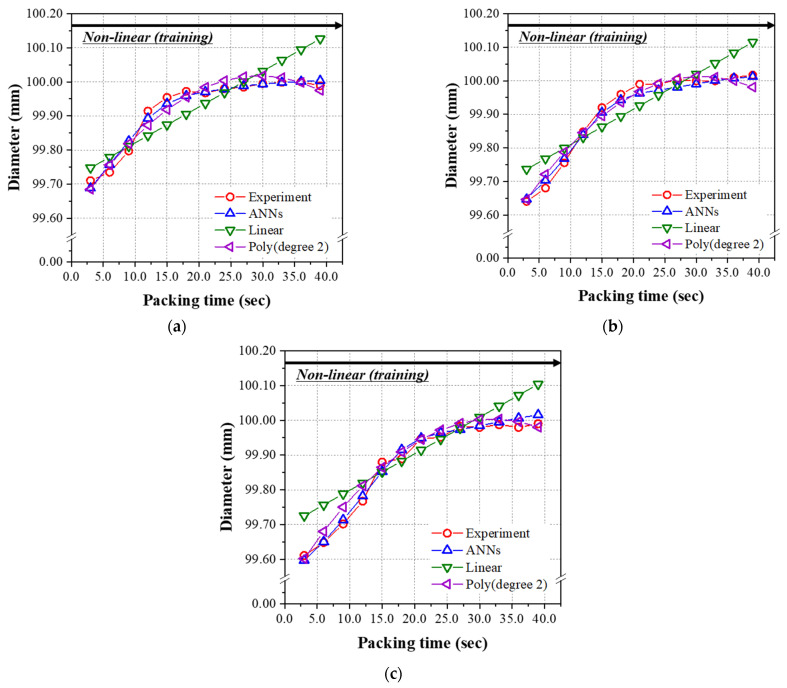
Predicted diameters of the nonlinear group from Table A2 using the models learned by the nonlinear group (packing time was 3.0–39.0 s). Melt temperatures were (**a**) 200 °C, (**b**) 220 °C, and (**c**) 240 °C.

**Figure 14:**
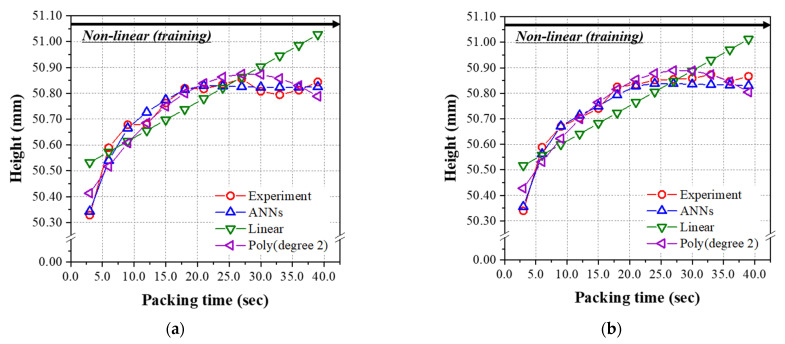

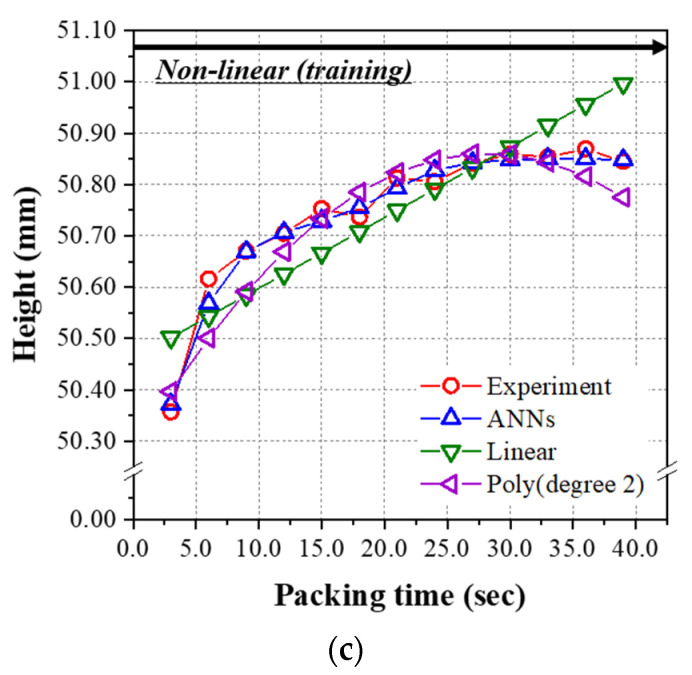
Predicted heights of the nonlinear group from Table A2 using the models learned by the nonlinear group (packing time was 3.0–39.0 s). Melt temperatures were (**a**) 200 °C, (**b**) 220 °C, and (**c**) 240 °C.

In the analysis of the nonlinear group, the predicted results of three models were graphically shown for the data sets in Table A2 as a function of the packing time up to 39.0 s. The previous test data set (#29, 34, 39, 44, 49, 59, 71,74, and 89) was substituted for the data set, including all the packing times (#51~89). Figure 12a–c, performed at 200 °C, 220 °C, and 240 °C, respectively, show the prediction and experimental results of the final mass as a function of the packing time for the linear range of 3.0~39.0 s. As seen in Table 13, the RMSE values of the mass for the ANN model of 1.709 × 10^−2^, and second-order polynomial regression of 2.105 × 10^−2^, are low enough to give an excellent prediction. As can be seen in the figures, the performance of the linear regression model is very poor and the RMSE value is very high. Similar to the results obtained from the test data set given in Table 12, the ANN model has the minimum and lowest RMSE among the three models for the diameter and height shown in Table 13. Figure 13 and Figure 14 show the prediction and experimental results for diameter and height as a function of the packing time. From the results of the data set in the nonlinear range for packing time (#51~89) of 3.0 s~39.0 s, the prediction performance of the final ANN model was the best among the three models. While there is a nonlinear relationship between input and output parameters, ANN is the best choice from our limited data sets.

## 5. Conclusions

Based on the results of the preliminary experiment, the data sets used in the present study were divided into two groups. One showed the linear relation between the mass of the final product and the range of packing time (linear relation group), and the other showed clear nonlinear relation (nonlinear relation group). For convenience, the linear relation group was specified to have the packing time less than or equal to 18.0 s. In other words, the range of the packing time was 3.0 s~18.0 s. The nonlinear relation group includes the data sets having a packing time up to 39.0 s, i.e., the input range of the packing time was 3.0 s~18.0 s.

ANN, linear regression, and second-order polynomial regression models were constructed for the linear and nonlinear groups, respectively. Furthermore, the performance of each predictive model and their ability to represent the relationships between parameters were compared. For the linear relation group, the prediction performance of the ANN model was relatively better than that of the linear regression and second-order polynomial regression models. However, all three models showed low RMSE, while the relation between the mass and the packing time showed high linearity. For the nonlinear relation group, the predicted results of the ANN model constructed in the present study were much better than that of the linear regression and second-order polynomial regression models. The ANN model might be the best method for predicting data concerning the relationship between input and output parameters, i.e., the range of input parameters including the nonlinear zone.

From the analysis of our specific data sets in the present study, ANN might be a better choice than linear or second-order polynomial regression if the data set has the characteristic of both linear and nonlinear. The results of this study might be a useful reference for future studies applying the ANN to the injection molding industry.

## Figures and Tables

**Table 1:** Previous research on ANNs applied to the injection molding process [7,8,9,10,11,12,13,14,15,16,17].

Author	Product	Input Parameters	Output Parameters	The Number of Hidden Layers	The Number of Neurons per Hidden Layers
Ozcelik, B et al. [7]	Thin shell part (CAE)	5 (Mold Temp., Melt Temp., Packing pressure, Packing time, Cooling time)	1 (Warpage)	2 hidden layers	9 (1st)–9 (2nd)
Yin, F et al. [8]	Automobile glove component (CAE)	5 (Mold Temp., Melt Temp., Packing pressure, Packing time, Cooling time)	1 (Warpage)	2 hidden layers	20 (1st)–20 (2nd)
Yang, D. C. et al. [9]	Cup (experiment)	10 (Melt Temp., Mold Temp., Injection speed, V/P switchover pressure, Packing pressure, Packing time, Cooling time, Back pressure, Plastification speed, Suck back)	1 (Mass)	2 hidden layers	43 (1st)–40 (2nd)
Lee, C.H et al. [10]	36 different products (CAE, experiment)	9 (Overall volume, Cavity volume, Overall surface area, Cavity surface area, Filling time, Melt Temp., Mold Temp., Packing pressure, Packing time)	1 (Weight)	2 hidden layers	28 (1st)–28 (2nd)
Gim, J. et al. [11]	Spiral (experiment)	10 (Time and pressure value from sensor)	1 (Part weight)	1 hidden layer	8
Abdul, R et al. [12]	Tensile specimens (experiment)	3 (Injection speed, Holding time, Cooling time)	2 (Length shrinkage, Width shrinkage)	1 hidden layer	4 (1st)
Heinisch, J et al. [13]	Plate (CAE)	6 (Mold Temp., Melt Temp., Injection time, Packing pressure, Packing time, Cooling time)	3 (Weight, length, width)	1 hidden layer	5 (1st)
Ke, K. C. et al. [14]	IC tray (experiment)	1~11 (Combinations of 11 pressure sensor signal)	3 points of width	1 hidden layer	1~33 (1st)
Huang, Y. M. et al. [15]	Circle plate (CAE)	5 (Injection speed, Packing time, Mold Temp., Melt Temp.)	3 (Injection pressure, Cooling time, Z shrinkage)	2 hidden layers	7 (1st)–3 (2nd)
			5 (Injection pressure, Cooling time, X, Y, Z shrinkage)	2 hidden layers	11 (1st)–7 (2nd)
Moayyedian, M. et al. [16]	Circle plate (CAE)	4 (Filing time, Cooling time, Packing time, Melt temperature)	3 (Short shot, Shrinkage rate, Warpage)	Not mentioned	Not mentioned
Yang, D. C. et al. [17]	LEGO (experiment)	8 (Melt Temp., Mold Temp., Injection speed, Packing pressure, Packing time, Cooling time, Back pressure, Screw speed)	5 (Mass, Pressure at the end of fill, X, Y, Z Length)	1 hidden layer	11 (1st)

**Table 2:** General properties of the polypropylene (PP) used in this study (LUPOL GP1007F, LG Chemical Co., Ltd.).

Properties		Standard	Condition	Unit	Value
Physical	Specific gravity	ASTM D792	-	-	0.94
	Melt flow rate	ASTM D1238	230 °C, 2.16 kg	g/10 min	13.0
Mechanical	Tensile strength (3.2 mm)	ASTM D638	50 mm/min	kgf/cm^2^	270
	Flexural strength (6.4 mm)	ASTM D790	10 mm/min	kgf/cm^2^	360
Thermal	Heat deflection Temp. (6.4 mm)	ASTM D648	4.6 kg	°C	125

**Table 3:** Specifications of the injection molding machine (LGEII-150, LSMtron).

Item	Value	Unit
Clamping force	150	ton
Screw diameter	32.0	mm
Max. injection speed	1000	mm/s
Max. injection pressure	3500	bar
Max. injection stroke	120	mm

**Table 4:** Process conditions and levels for the experiment.

Conditions	Level 1	Level 2	Level 3
Melt temperature (°C)	200	220	240
Mold temperature (°C)	40	50	60
Injection speed (mm/s)	40	70	100
Packing pressure (bar)	150	200	250
Packing time (s)	6.0	12.0	18.0
Cooling time (s)	38	48	58

**Table 5:** Injection molding conditions of linear relation group.

Exp. No.	Melt Temperature (°C)	Mold Temperature (°C)	Injection Speed (mm/s)	Packing Pressure (bar)	Packing Time (s)	Cooling Time (s)	Note
1	200	40	40.0	150	6.0	38	L27
2	200	40	40.0	150	12.0	48	L27
3	200	40	40.0	150	18.0	58	L27
4	200	50	70.0	200	6.0	38	L27
5	200	50	70.0	200	12.0	48	L27
6	200	50	70.0	200	18.0	58	L27
7	200	60	100.0	250	6.0	38	L27
8	200	60	100.0	250	12.0	48	L27
9	200	60	100.0	250	18.0	58	L27
10	220	40	70.0	250	6.0	48	L27
11	220	40	70.0	250	12.0	58	L27
12	220	40	70.0	250	18.0	38	L27
13	220	50	100.0	150	6.0	48	L27
14	220	50	100.0	150	12.0	58	L27
15	220	50	100.0	150	18.0	38	L27
16	220	60	40.0	200	6.0	48	L27
17	220	60	40.0	200	12.0	58	L27
18	220	60	40.0	200	18.0	38	L27
19	240	40	100.0	200	6.0	58	L27
20	240	40	100.0	200	12.0	38	L27
21	240	40	100.0	200	18.0	48	L27
22	240	40	40.0	250	6.0	58	L27
23	240	50	40.0	250	12.0	38	L27
24	240	50	40.0	250	18.0	48	L27
25	240	60	70.0	150	6.0	58	L27
26	240	60	70.0	150	12.0	38	L27
27	240	60	70.0	150	18.0	48	L27
28	214	55	82.7	204	16.3	52	Random
29	204	44	43.4	202	13.9	41	Random
30	203	46	93.6	205	13.7	45	Random
31	202	54	83.4	213	6.6	48	Random
32	206	43	61.6	221	6.9	39	Random
33	212	44	53.3	240	17.0	52	Random
34	212	51	90.8	224	6.1	48	Random
35	200	52	50.0	215	17.6	39	Random
36	229	51	46.2	153	11.7	45	Random
37	228	49	53.2	217	12.3	58	Random
38	222	51	63.7	167	8.7	51	Random
39	219	50	41.4	156	16.3	52	Random
40	228	46	96.5	154	16.7	57	Random
41	228	46	62.5	191	10.9	46	Random
42	219	42	98.4	237	17.9	41	Random
43	220	43	55.8	241	14.8	44	Random
44	233	42	50.8	198	13.5	55	Random
45	238	53	41.6	221	17.2	40	Random
46	234	48	68.2	222	8.8	41	Random
47	233	44	84.9	171	6.7	55	Random
48	234	43	56.9	176	11.1	48	Random
49	239	49	41.2	234	8.6	52	Random
50	240	49	76.1	241	6.4	51	Random

**Table 6:** Injection molding conditions of nonlinear relation group.

Exp. No.	Melt Temperature (°C)	Mold Temperature (°C)	Injection Speed (mm/s)	Packing Pressure (bar)	Packing Time (s)	Cooling Time (s)	Note
51– 63	200	50	70	200	3.0–39.0 (interval: 3)	38	Non-linear case
64– 76	220	50	70	200	3.0–39.0 (interval: 3)	38	Non-linear case
77– 89	240	50	70	200	3.0–39.0 (interval: 3)	38	Non-linear case

**Table 7:** Ranges of hyper-parameters obtained by hyper-band technique [25].

Hyper-Parameters	Range	Note
Seed number	0–50	Step size was 1
Batch size	16, 32, 64, …	Increased in multiples of 2 until it could cover the number of learning data
Optimizer	Adams [26]	Fixed
Learning rate	0.0001–0.01 [26]	Step size was 0.0001
Beta 1	0.1–1.0 [26]	Step size was 0.1
Bata 2	0.9, 0.99, 0.999, 0.999 [26]	-
Number of hidden layers	1–5 (shared layers) 1 (task-specific layer)	Step size was 1 (task-specific layer was fixed as one layer)
Number of neurons	3–18	Step size was 1
Initializer	He normal (hidden layer) Xavier normal (output layer)	-
Activation function	Elu (hidden layer) Linear (output layer)	-
Drop number	0.0–0.4	Step size was 0.1
Coefficient of batch normalization	0.001, 0.01, 0.1	-

**Table 8:** Optimized hyper-parameters for the linear relationship group.

Hyper-Parameters	Value
Seed number	16
Batch size	16
Optimizer	Adams
Learning rate	0.0069
Beta 1	0.6
Beta 2	0.9
Number of hidden layers	3 (shared layers) 1 (specific-task layer)
Number of neurons	17–13–13 (shared layers) 13 (specific-task layers for mass) 9 (specific-task layers for diameter) 8 (specific-task layers for height)
Initializer	He normal (hidden layers) Xavier normal (output layer)
Activation function	Elu
Drop number	0.0–0.2–0.2 (shared layers) 0.0 (specific-task layers for mass) 0.3 (specific-task layers for diameter) 0.3 (specific-task layers for height)
Coefficient of batch normalization	0.001 (mass), 0.01 (diameter), 0.001 (height)

**Table 9:** Root mean square errors (RMSEs) of normalized test data for prediction models learned by the linear relation group (packing time was 3.0–18.0 s).

Prediction Model	RMSE		
	Mass	Diameter	Height
ANN	$1.279\times 10^{-2}$	$6.283\times 10^{-2}$	$2.467\times 10^{-2}$
Linear regression	$1.440\times 10^{-2}$	$8.834\times 10^{-2}$	$4.860\times 10^{-2}$
Polynomial regression of degree 2	$1.317\times 10^{-2}$	$1.360\times 10^{-1}$	$3.362\times 10^{-2}$

**Table 10:** Root mean square errors (RMSEs) of normalized Table A2 (packing time was 3.0~18.0 s) for prediction models learned by the linear relation group (packing time was 3.0~18.0 s).

Prediction Model	RMSE		
	Mass	Diameter	Height
ANN	$1.871\times 10^{-2}$	$3.090\times 10^{-2}$	$3.925\times 10^{-2}$
Linear regression		$8.240\times 10^{-2}$	$7.821\times 10^{-2}$
Polynomial regression of degree 2	$9.294\times 10^{-3}$	$7.101\times 10^{-2}$	$5.218\times 10^{-2}$

**Table 11:** Optimized hyper-parameters for the nonlinear relationship group (packing time was 3.0~39.0 s).

Hyper-Parameters	Value
Seed number	35
Batch size	16
Optimizer	Adams
Learning rate	0.0073
Beta 1	0.5
Beta 2	0.9
Number of hidden layers	2 (shared layers) 1 (specific-task layer)
Number of neurons	6–5 (shared layers) 4 (specific-task layers for mass) 3 (specific-task layers for diameter) 4 (specific-task layers for height)
Initializer	He normal (hidden layers) Xavier normal (output layer)
Activation function	Elu
Drop number	0.0–0.0 (shared layers) 0.2 (specific-task layers for mass) 0.1 (specific-task layers for diameter) 0.0 (specific-task layers for height)
Coefficient of batch normalization	0.001 (mass), 0.01 (diameter), 0.001 (height)

**Table 12:** Root mean square errors (RMSEs) of normalized test data for prediction models learned by the nonlinear relation group (packing time was 3.0–39.0 s).

Prediction Model	RMSE		
	Mass	Diameter	Height
ANN	$1.966\times 10^{-2}$	$5.453\times 10^{-2}$	$2.917\times 10^{-2}$
Linear regression	$8.427\times 10^{-2}$	$1.283\times 10^{-1}$	$9.514\times 10^{-2}$
Polynomial regression of degree 2	$2.702\times 10^{-2}$	$9.848\times 10^{-2}$	$4.436\times 10^{-2}$

**Table 13:** Root mean square errors (RMSEs) of normalized Table A2 (packing time was 3.0~39.0 s) for prediction models learned by the nonlinear group (packing time was 3.0~39.0 s).

Prediction Model	RMSE		
	Mass	Diameter	Height
ANN	$1.709\times 10^{-2}$	$2.871\times 10^{-2}$	$2.578\times 10^{-2}$
Linear regression	$1.096\times 10^{-1}$	$1.193\times 10^{-1}$	$1.084\times 10^{-1}$
Polynomial regression of degree 2	$2.105\times 10^{-2}$	$4.273\times 10^{-2}$	$5.081\times 10^{-2}$

## Data Availability

Not applicable.

## Appendix A

**Table A1:** Product qualities for injection molding conditions with linearity.

No.	Mass (g)	Diameter (mm)	Height (mm)	Note	No.	Mass (g)	Diameter (mm)	Height (mm)	Note
1	54.05	99.77	50.48	L27	26	54.08	99.85	50.50	Random
2	55.89	99.88	50.72	L27	27	55.29	99.93	50.68	Random
3	56.96	99.88	50.82	L27	28	56.16	99.91	50.78	Random
4	54.33	99.73	50.59	L27	29	56.22	99.92	50.79	Random
5	55.72	99.90	50.73	L27	30	56.05	99.93	50.78	Random
6	57.17	99.95	50.88	L27	31	54.11	99.79	50.51	Random
7	54.13	99.74	50.52	L27	32	54.44	99.83	50.56	Random
8	55.69	99.92	50.77	L27	33	57.07	100.00	50.92	Random
9	57.15	100.00	50.92	L27	34	53.96	99.73	50.49	Random
10	54.24	99.69	50.57	L27	35	57.06	99.93	50.90	Random
11	55.99	99.94	50.82	L27	36	54.68	99.84	50.59	Random
12	57.31	100.02	50.95	L27	37	55.49	99.86	50.74	Random
13	53.22	99.76	50.43	L27	38	54.07	99.79	50.51	Random
14	54.86	99.90	50.61	L27	39	56.02	99.99	50.75	Random
15	55.97	99.91	50.74	L27	40	56.04	99.96	50.78	Random
16	53.75	99.77	50.45	L27	41	54.92	99.89	50.65	Random
17	55.25	99.88	50.67	L27	42	56.93	100.01	50.92	Random
18	56.22	99.89	50.77	L27	43	56.53	100.02	50.85	Random
19	53.38	99.64	50.45	L27	44	55.58	99.96	50.75	Random
20	54.87	99.92	50.67	L27	45	56.12	100.02	50.81	Random
21	56.30	100.02	50.86	L27	46	54.31	99.81	50.56	Random
22	53.89	99.71	50.51	L27	47	53.52	99.79	50.43	Random
23	55.22	99.94	50.73	L27	48	54.73	99.94	50.61	Random
24	56.60	100.05	50.92	L27	49	54.47	99.80	50.61	Random
25	52.64	99.66	50.26	L27	50	53.80	99.78	50.52	Random

**Table A2:** Product qualities for injection molding conditions with nonlinearity according to packing time.

No.	Mass (g)	Diameter (mm)	Height (mm)	No.	Mass (g)	Diameter (mm)	Height (mm)
51	53.46	99.71	50.33	71	57.30	99.99	50.85
52	54.33	99.73	50.59	72	57.32	100.00	50.85
53	55.08	99.80	50.68	73	57.38	100.00	50.86
54	55.74	99.91	50.68	74	57.41	100.00	50.87
55	56.37	99.95	50.76	75	57.44	100.01	50.85
56	56.97	99.97	50.82	76	57.48	100.02	50.87
57	57.27	99.97	50.82	77	52.56	99.61	50.36
58	57.34	99.98	50.83	78	53.46	99.65	50.52
59	57.35	99.98	50.86	79	54.22	99.70	50.67
60	57.38	99.99	50.81	80	54.89	99.77	50.70
61	57.40	100.00	50.79	81	55.51	99.88	50.75
62	57.46	100.00	50.81	82	56.13	99.89	50.74
63	57.46	99.99	50.84	83	56.72	99.95	50.81
64	53.03	99.64	50.34	84	57.14	99.95	50.81
65	53.92	99.68	50.59	85	57.31	99.98	50.84
66	54.68	99.76	50.67	86	57.35	99.98	50.86
67	55.45	99.85	50.70	87	57.39	99.99	50.85
68	56.08	99.92	50.74	88	57.42	99.98	50.87
69	56.64	99.96	50.83	89	57.48	99.99	50.85
70	57.16	99.99	50.83				
